# Supplementary material for: Empirical methods for controlling false positives and estimating confidence in ChIP-Seq peaks
Source: BMC Bioinformatics. 2008 Dec 5;9:523. doi: 10.1186/1471-2105-9-523 (PMC2628906; doi:10.1186/1471-2105-9-523)
Supplement: Additional File 1 — A variety of html documents from the USeq web site detailing the available applications, their best usage, output file type descriptions, command line menus, etc. [file 1471-2105-9-523-S1.zip › USeqUserGuides/JavaDoc.zip!/JavaDoc/gata/aligner/Blast.html]

Blast


|  |  |  |
| --- | --- | --- |
| **Overview** \| **Package** \| **Class** \| **Use** \| **Tree** \| **Deprecated** \| **Index** \| **Help** | |  |
| **PREV CLASS**   **NEXT CLASS** | **FRAMES**    **NO FRAMES**     **All Classes** |  |
| SUMMARY: NESTED \| FIELD \| CONSTR \| METHOD | DETAIL: FIELD \| CONSTR \| METHOD |  |


---


## gata.aligner Class Blast

```
java.lang.Object
  gata.aligner.Blast
```

---

``` public class Blast extends java.lang.Object ```

**Author:**
:   nix
    Class to fire and fetch the ncbi bl2seq program.

---

| **Field Summary** | |
| --- | --- |
| `AlignParams` | `ap` |


**Constructor Summary**

`Blast(AlignParams AP)`


| **Method Summary** | |
| --- | --- |
| `java.lang.String[]` | `blastIt(java.lang.String compSeqFileName)` |
| `java.util.ArrayList` | `parseBlast(java.lang.String[] data)` |
| `static java.util.ArrayList` | `parseLine(java.lang.String line, java.util.regex.Pattern pat)` |

**Methods inherited from class java.lang.Object**

`equals, getClass, hashCode, notify, notifyAll, toString, wait, wait, wait`

**Field Detail**

### ap

```
public AlignParams ap
```


**Constructor Detail**

### Blast

```
public Blast(AlignParams AP)
```


**Method Detail**

### blastIt

```
public java.lang.String[] blastIt(java.lang.String compSeqFileName)
```

---


### parseBlast

```
public java.util.ArrayList parseBlast(java.lang.String[] data)
```

---


### parseLine

```
public static java.util.ArrayList parseLine(java.lang.String line,
                                            java.util.regex.Pattern pat)
```


---


|  |  |  |
| --- | --- | --- |
| **Overview** \| **Package** \| **Class** \| **Use** \| **Tree** \| **Deprecated** \| **Index** \| **Help** | |  |
| **PREV CLASS**   **NEXT CLASS** | **FRAMES**    **NO FRAMES**     **All Classes** |  |
| SUMMARY: NESTED \| FIELD \| CONSTR \| METHOD | DETAIL: FIELD \| CONSTR \| METHOD |  |


---
